# Effects of lemon oil-based nanoemulsion on sensory and microbiological quality of vacuum packed raw versus steam cooked trout (*Oncorhynchus mykiss*, Walbaum 1792) stored at + 4 ± 2 °C

**DOI:** 10.1007/s13197-025-06352-6

**Published:** 2025-06-16

**Authors:** Bengunur Corapci, Demet Kocatepe, Can Okan Altan, Zafer Ceylan, Bayram Kostekli, Hulya Turan

**Affiliations:** 1 Fishery Faculty, Department of Fish Processing Technology, Sinop University, Sinop, Turkey; 2 Science Faculty, Department of Molecular Biology and Genetics/Biotechnology, Bartın University, Bartın, 74000 Turkey

**Keywords:** Nanoemulsion, Lemon oil, Trout, Steam cooking, Vacuum package, Microbiological spoilage, Sensory evaluation

## Abstract

The changes in TVB-N (Total Volatile Base Nitrogen) content, sensory and microbiological properties of raw and steamed trout cubes treated with lemon oil nanoemulsion (LON) were examined for 30 days at 4 ± 2 °C and the shelf life was determined. RT (raw trout), RTL (raw trout treated with LON), ST (steamed trout), and STL (steamed trout treated with LON) groups were assigned. Zeta size, polydispersity index and zeta potential values of fabricated nanoemulsion were determined as equal to 197.067 nm, 0.217, and − 3.56 mV, respectively. The numbers of Total Mesophilic Aerobic Bacteria (TMAB), Total Psychrophilic Aerobic Bacteria (TPAB), Total Yeast and Mold (TYM), Total Coliform Bacteria (TCB), and Total Anaerobic Bacteria (TAB) were significantly decreased in the steam cooking and LON treated groups.The increase in the TVB-N amount was limited in the samples treated with nanoemulsion, and STL samples showed better sensory quality. As a result, nanoemulsification and steam cooking application increased the sensory and microbiological quality of trout and extended its shelf life.

## Graphical abstract

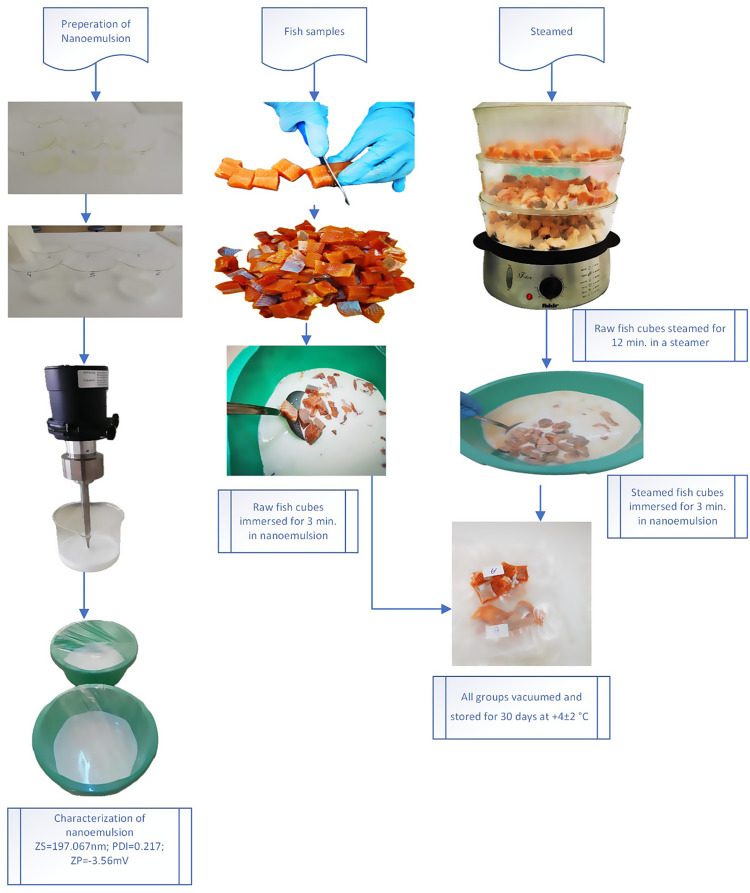

## Introduction

Food or food safety is a unique issue for consumers and food producers. In addition, new food products especially related to ready-to-eat products, are receiving great attention in the food industry. This type of product offers some advantages to workers who have little time to prepare food for dinner and also saves time at home or in the kitchen. In this sense, some cooking techniques such as sous vide which could be combined with other food preservation techniques are effectively used for this goal. This combination provided better sensory preferences while the nano-combination limited rapid microbial spoilage in the salmon samples stored at 4 °C (Ceylan et al. 2022). In particular, as shown from the given study, some quality parameters (TVB-N, microbial quality indices, pH etc.) could play a key role in determining the quality changes after the process or depending on the shelf life. In addition to widely used conventional methods, recently, novel approaches based on nanotechnology were tried directly or indirectly on food materials. These include, nanofibers, nanoemulsions, nanoparticles, nano-scale nanoencapsulated materials, nanoliposomes and nanowhiskers which are currently being used by researchers around the world to improve the quality of food materials. In addition to nanoencapsulation of probiotics (*L. rhamnosus*,* L. reuteri*,* Streptococcus thermophilus*) (Ocak and Demirci 2023), thiamine-loaded nanofibers (Yaman et al. 2022), and seaweed-based nanoliposomes (Mousavipour et al. 2021), particularly nanoemulsions (Durmus et al. 2020) obtained from different citrus have been practiced to provide the food stability. In this regard, lemon essential oil, which mainly contains limonene, terpenes and citral, can effectively inhibit the growth of most microorganisms, as reported by Zhang et al. (2022)d nchez-González et al. (2011). Unlike the mentioned benefits of lemon for human consumption, there are also limited disadvantages of consuming lemon or lemon juice. As widely known, the ingestion of lemon by consumers with gastric problems can exacerbate the stomach problem, and, so, in this regard, decreasing the amount of lemon using nanotechnology applications could be evaluated as a better solution. As is known, nanotechnology provides a larger contact area on the surface of the food material, allowing better solutions to be achieved with less material compared to micro-sized materials (Tatlisu & Torusdag, 2023; Ceylan et al. 2020b).

The aim of the present study was to produce smaller size nanoemulsions based on lemon essential oils and to characterize them. After the determination of the characteristic parameters (PDI, zeta size and zeta potential), the second objective was to reveal the possible effects on the microbial, chemical, physical and sensory quality of lemon essential oil-based nanoemulsions for ready-to-eat fish meat samples. With the results, the ultimate goal was to show that the study based on nanoscience and ready-to-eat foods could play a pioneering role for further applications of nanomaterials in the food industry.

## Materials and methods

### Materials

The deceased rainbow trout (*Oncorhynchus mykiss*, Walbaum 1792) used for the present research were purchased from a local market in Türkiye. A total of 25 kg trout was used (15 pieces). The fish samples were transferred to the Fisheries Faculty Processing and Quality Control Laboratory in styrofoam boxes including ice two hours after the harvest. The mean length and weight of the fish were determined to be 47.95 ± 0.77 cm and 1659.05 ± 70.87 g, respectively. Then the fish skin was removed and the body was cut into cubes. The cube dimensions of the fish meat samples were 2.73 ± 0.08*2.31 ± 0.07*1.73 ± 0.09 cm and the mean cube weight was 13.47 ± 0.57 g (*n* = 20).

## Methods

### Production of nanoemulsion

14% lemon oil (Uğurluoğlu LTD. ŞTİ. Akşehir, Konya), 3% ethanol (Supelco 1.00983.2511), 3% Tween 80 (ISOLAB, 960.161.1000) (GRAS = generally recognized as safe-harmless) were reported to be used by Hamouda et al. (1999). This mixture was left at 86 °C for 1 h and then cooled to room temperature. Later, 80% distilled water was added and homogenized in an ultrasound device (Bandelin, Sonopuls HD 2200) for 15 min. The prepared nanoemulsions were sterilized under a UV lamp (Bil-Ser, Turkey) for 20 min.

### Nanoemulsion treatment with fish meat

Fish cubes were assigned to four groups represented by their initials: RT (Raw trout cubes), RTL (Raw trout cubes treated with lemon oil nanoemulsion), ST (Steamed trout cubes), and STL (Steamed trout cubes treated with lemon oil nanoemulsion). The raw trout fish meat cubes in group RT were vacuum-packed without any treatment. In the RTL group, the fish meat cubes were immersed in the prepared nanoemulsion solution, left for 3 min, filtered and then vacuum packed. In ST group, the fish meat cubes were cooked for 12 min in a steamer (Fakir Tolero, 1,000 W, 9.9 L capacity, Stuttgart, Germany). After cooling to room temperature, they were vacuum packed. In the STL group, the fish meat cubes were cooked in a steamer (Fakir Tolero, 1,000 W, 9.9 L capacity, Stuttgart, Germany) for 12 min. Then they were immersed in the prepared nanoemulsion solution and kept for 3 min, filtered out and then vacuum-packed. Finally, each group is represented by 200 g fish in the vacuumed package (Abant Group, MG42, Hellas, Sydney, Australia). A total of 11 fish meat samples were collected and the study was carried out in two replicates. After vacuum packaging, all group samples were stored at 4 ± 2 °C for 30 days.

### Characterization of nanoemulsion

The zeta potential (ZP), mean zeta size (ZS), and polydispersity index (PDI) of the obtained nanoemulsion were determined using a Dynamic Light Scattering (DLS) at 25 °C (Malvern Instruments, Worcestershire, UK). An average of 30 measurements was taken for each sample.

### Microbiological analysis

#### Total mesophilic aerobic Bacteria

Total Mesophilic Aerobic Bacteria (TMAB log CFU/g) was determined according to the instructions of Halkman (2005). 10 g of sample for each group were homogenized separately for 150 s in a stomacher (IU Instruments, Spain) with 90 mL of 0.1%. peptone water and serial dilutions of 10 to 10^6^ were prepared for each sample. All diluted samples were placed on plate count agar (PCA, Merck) and incubated (at 35 °C for 24 h) to determine TMAB counts.

#### Total psychrophilic aerobic Bacteria

In addition to the methodology given above for TMAB, serial dilutions up to 10^6^ (control and treated samples) were added to PCA and then incubated at 7 °C for 10 days to produce TPB according to the method described as log CFU/g by Halkman (2005).

#### Total yeast and mold

The Potato Dextrose Agar (PDA, Biolife 4019352) at 10^6^ was poured into sterile dishes and incubated at 25 °C for 5 days. The results were reported as log CFU/g, as described by Halkman (2005).

#### Total coliform bacteria

The medium prepared using Violet Red Bile Agar (VRBA, Biolife 4021852) was poured into sterile dishes and incubated at 37 °C for 1 day. The colonies formed on the petri dish at the end of the incubation were calculated and presented as log CFU/g, as reported by Halkman (2005).

#### Total anaerobic bacteria

The Reinforced Clostridial Medium Agar (RCM, Biolife 4013032) medium was used for total anaerobic bacteria counts. The petri dishes were incubated in an anaerobic incubator at 35–37°C for 24 h. All colonies growing on the medium were counted as ‘anaerobic bacteria’ (Halkman 2005).

### Total volatile base nitrogen (TVB-N)

TVB-N analyzes of the samples were performed using the Lücke-Geidel method modified by Antonacopoulas (Varlık et al., 1993). 1 g of MgO, a few drops of silicone oil and 100 ml of distilled water were added to 10 g of homogenized sample and placed in a glass flask. 10 ml of 3% boric acid, 8 drops of tashiro indicator and 100 ml of distilled water were added into the erlenmeyer, which was used as a titration vessel in a separate place. After the glass balloon and flask containing the sample were placed to receive the distillate, they were subjected to distillation for 15–20 min. The resulting distillate was titrated with 0.1 N HCl until the color changed from green to pink. The results for each group sample were expressed as mg TVB-N/100 g.

### Sensory analysis

Ten grams of fish samples for each group were presented to the panelists on white porcelain plates and the appearance, taste, odor, texture, and overall acceptability of all groups were assessed using the method given by York and Sereda (1994). Five experienced panelists from the Faculty of Fisheries participated in the sensory analyses. Our faculty does not request ethical permission from us when performing sensory analyses. In addition, participants gave informed consent via the statement “I am aware that my responses are confidential, and I agree to participate in this survey” where an affirmative reply was required to enter the survey. They were able to withdraw from the survey at any time without giving a reason. The products tested were safe for consumption. In this sense, they scored the samples on a 5-point hedonic scale, as follows: 5, I like it very much, 4, I like it, 3, I like it moderately, 2, I don’t like it (limit value), and 1, I don’t like it at all. Products with ≤ 2 points are considered as non-consumable (*n* = 20).

### Statistical analysis

All data obtained were analyzed using the Microsoft Office Excel 2020 program (means and standard errors) and performed with one-way analysis of variance (ANOVA) and Tukey test using Minitab 17 (Minitab Inc., State College, PA, USA) (*p* < 0.05). Origin SR1 (2022, MA USA) was used to perform principal component analysis (PCA) result plots.

## Result and discussion

### Characterization of nanoemulsion

#### Zeta size

The mean ZS value of the nanoemulsion was defined as 197.067 ± 4.31 nm. Droplet size can lead to resistance to separation, flocculation and also coalescence by gravity. In addition to the type of material, the size of nanoscale material also plays an important role and can have an antimicrobial effect on food. In addition to the type of material, the nanoscale material size also plays an important role and can have an antimicrobial effect on food. In this regard, the use of rosemary nanoemulsion with a diameter of 158.3 nm could effectively limit the rapid TMAB growth in food samples, as reported by Ceylan et al. (2020a). Bhargava et al. (2015) and Meral et al. (2019) found that the average values of the thyme oil nanoemulsion could be determined in the range of 148 and 219 nm. In the present study, the nanoemulsion obtained from lemon and used for food combination had smaller droplet size, which could affect the antibacterial property of nanoemulsions treated with fish fillets.

#### Polydispersity index

The average value of the PDI of the obtained nanoemulsions was found to be 0.217 ± 0.025. The aim of the PDI measurement was to determine a measure of the size distribution. The PDI values of nanoemulsions obtained from different bioactive materials such, as thyme and eugenol, were in the range of 0.082 and 0.24 (Majeed et al. 2016; Meral et al. 2019). In the present study, the prepared nanoemulsions showed homogeneity. In another study, Ceylan et al. (2020a) reported an interaction between microbiological limitation and homogeneity properties of nanoemulsions on food materials.

#### Zeta potential

The ZP value measured to determine the electrokinetic potential in the colloidal dispersion of the obtained nanoemulsions was − 3.56 ± 0.61 mV. ZP value of nanomaterial can be determined as positive or negative mV. In this context, Watanabe et al. (2005) found that nanoparticles made from chitosan had a -3 mV ZP value. In this sense, all the results reported above show that ZP value of the nanoemulsion showed good stability here. On the other hand, there is a good correlation between ZP value, and the pH of the solution used to prepare the nanoemulsion. Therefore, in the present study, the pH of the solution used for the nanoemulsion was set at 4.23 and this result can affect the ZP value of the prepared nanoemulsion.

### Microbiological analyses

#### Total mesophilic aerobic Bacteria

**Fig. 1:**
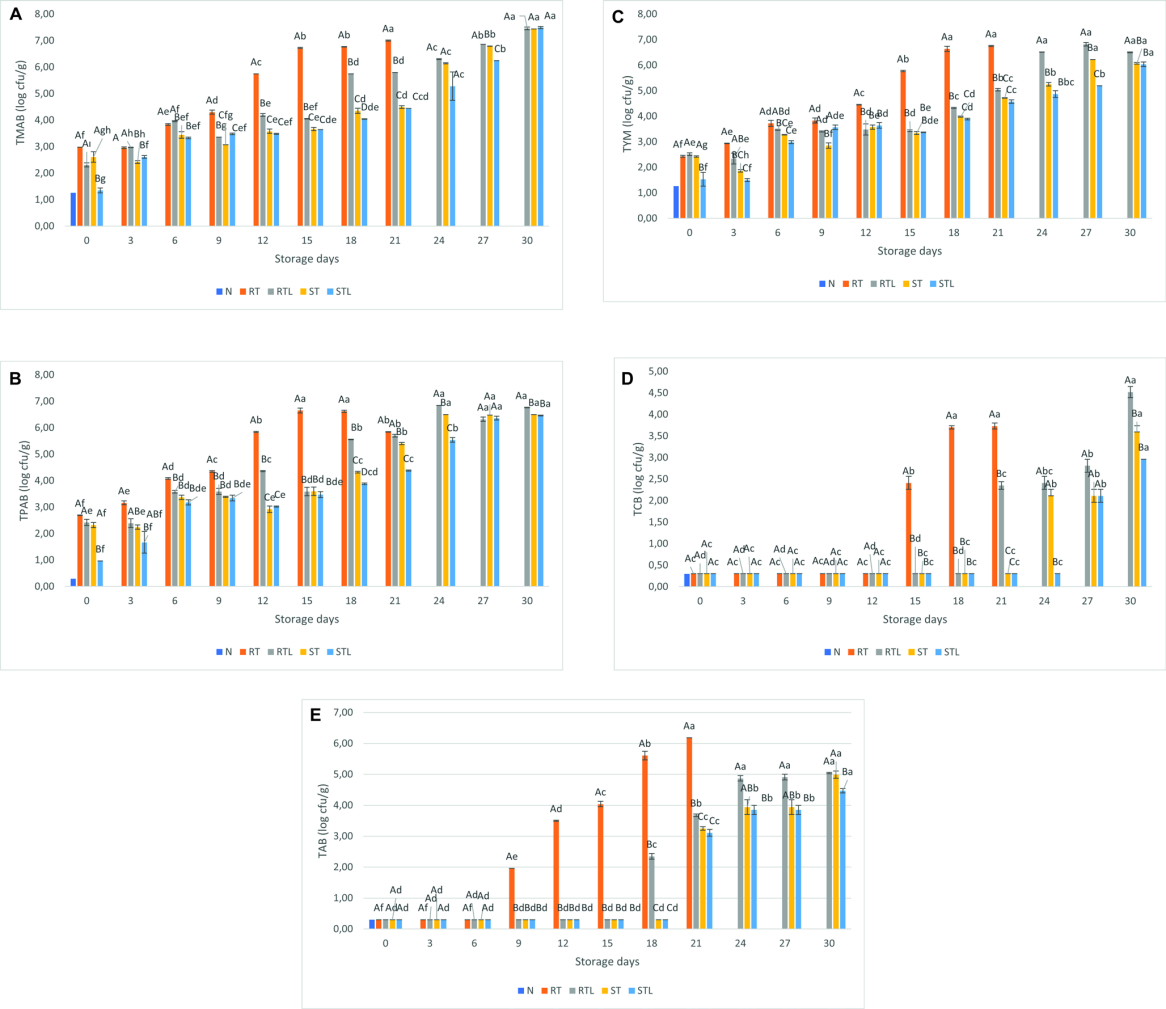
Total mesophilic aerobic bacteria (TMAB) (**A**), Total psychrophilic aerobic bacteria (TPAB) (**B**), Total yeast-mold (TYM) (**C**), Total coliform bacteria (TCB) (**D**), Total anaerobic bacteria (TAB) values of raw or steamed trout cubes treated/not treated with lemon oil nanoemulsion **N**: Nanoemulsion, **RT**: Raw trout cubes, **RTL**: Raw trout cubes treated with lemon oil nanoemulsion, **ST**: Steamed trout cubes, **STL**: Steamed trout cubes treated with lemon oil nanoemulsion

Although the effect of heat treatment on microorganisms varies according to the type of microorganism, it generally depends on changes in cell structure. Heat treatment affects the microbial cell wall, cell membrane, nucleic acids, structural enzymes and proteins in the cell. As a result of heat treatment, some of the microorganisms are irreversibly killed, while others may be damaged and survive. The damaged microorganisms can develop and cause problems as a result of the processed foods not being stored under appropriate conditions (Erkmen 2010). The TMAB counts of all samples are shown in Fig. 1.A. Furthermore, the initial TMAB count of the nanoemulsion was determined to be 1.26 log CFU/g. The initial TMAB count of the RT, RTL, ST, and STL samples were 2.98, 2.31, 2.61, and 1.35 log CFU/g, respectively. Specifically, as seen in the results, this nanoapplication had a higher effect on RTL and STL samples. In this regard, it can be concluded that the cooking process (heat treatment) and nanoemulsion treatment showed a hurdle effect on TMAB count limitation (*p* < 0.05). Furthermore, on the 21st day of cold storage, although the RT samples reached 7 log CFU/g, the TMAB count of the RTL samples was determined to be 5.80, and also the TMABc of the steamed samples were 4.49 and 4.44 log CFU/g, respectively. It is known that several studies are available in the literature on cooking and nanotreatment, but the result of the present study is highly promising. The combined effect of steaming and lemon oil nanoemulsion can be clearly seen on the 27th day of storage (*p* < 0.05). On the last day of storage (day 30), values above 7 log cfu/g and microbial spoilage were recorded in all groups. Pourhajibagher et al. (2022) showed that curcumin-nisin and PLA nanoparticles exhibited antibacterial effects on mesophilic microorganisms. So, from recently published studies, it appears that there is a growing trend on this issue. In our study, this limit was found to be higher than 3 log CFU/g for 21 days of storage compared to the control group. Ceylan et al. (2022) found that the nanofiber treatment of salmon meat samples cooked using the sous vide technique delayed TMAB growth up to 2.79 log CFU/g. By evaluating all the results, the present study revealed effective preservation with nanoemulsion for steamed fish fillets.

#### Total psychrophilic aerobic Bacteria

The TPAB counts of all samples are shown in Fig. 1.B. The initial TPAB count of the nanoemulsion was determined to be < 0.30 log CFU/g. The initial TPAB loads of the RT, RTL, ST, and STL samples were 2.69, 2.42, 2.32, and 0.96, respectively. As can be seen from the first analysis results, the application treated with STL samples in particular proved to be very effective (*p* < 0.05). During cold storage period STL, ST, and RTL samples were observed to have lower bacteria loads. This result showed that the combination of nanoemulsion with cooking limited TPB growth. Furthermore, on the 15th day of cold storage, the TPAB count of the RT samples was 6.65 log CFU/g, while the STL samples had a TPAB count of 3.47 log CFU/g. At the end of storage, the STL group was observed to have the lowest TPAB count. Previous studies on oils showed that curcumin, rosemary oil and other essential oils had potential antibacterial properties (Abdou et al. 2018; Pires et al. 2018). Durmus (2020) also found that the number of psychrophilic bacteria was lower in the fish samples treated with nanoemulsions compared to the control group. As is known, psychrophilic bacteria strains are defined as the main group of bacteria that cause spoilage in refrigerated fish meat samples. Therefore, this combination of treatment for limiting TPAB count growth in ready-to-eat samples can be evaluated as an alternative and effective application.

#### Total yeast and mold

The TYM counts of all samples are shown in Fig. 1.C. The TYM load of the nanoemulsion was determined to be 1.27 log CFU/g. In the present study, ready-to-eat fish products treated with lemon nanoemulsion followed by cooking had lower yeast and mold counts. Furthermore, this limit was set at 58.59% (6.64 to 3.89 log CFU/g) compared to the control sample on day 18th of the analysis period. The maximum TYM counts in RT, RTL, ST, and STL samples on day 21 were defined as 6.75, 5.04, 4.71, and 4.57 log CFU/g, respectively. Likewise, on the last day of storage (30th day), the lowest number of TYM was detected in the STL group.

Corapci (2022) reported that rosehip seed oil nanoemulsion was not significantly effective in reducing total yeast and mold counts. In another study, Zhao et al. (2023) reported that rosehip seed oil nanoemulsion application decreased the activation of TYM in sea bass. As evident from the results, the cooking method and nanoemulsion treatment effectively limited the rapid TYM growth in the samples. In addition, safety of fish raw materials (RTL sample) was ensured to obtain ready-to-eat samples and to make them usable for further food processes. As is well known, initial quality is considered as one of the most important factors in the production of healthy food. In this sense, the present nanotechnology-based approach can contribute to help the food safety of ready-to-eat samples.

#### Total coliform Bacteria

TCB counts of all samples are shown in Fig. 1.D. The TCB count of the nanoemulsion was determined to be < 0.30 log CFU/g. Likewise, the number of TCB was < 0.30 log CFU/g up to the 12th day of cold storage period. On the other hand, the coliform count began to increase on days 15, 18 and 21 and reached 3.73 log CFU/g. This case was also reported for RTL samples on 21st day of cold storage with a load of 2.35 log CFU/g. However, the number of coliform bacteria in both steamed groups (ST, STL), treated/untreated with nanoemulsion was found to be < 0.30 log CFU/g. On the last day of storage, coliform counts increased in all RTL, ST and STL groups and were determined to be 4.52 log CFU/g, 3.59 log CFU/g and 2.96 log CFU/g, respectively.

Cooking processes can be a very effective elimination of coliform bacteria strains, but some types of nanoemulsion such as rosehip seed oil nanoemulsion had no antibacterial effect on coliform bacteria in fish meat, as reported by Corapci et al. (2022). In the present study, the combination of cooking and nanoemulsion in fish meat was found to indicate a limiting effect on the growth of coliform bacteria.

#### Total anaerobic Bacteria

Vacuum packaging is known to promote the growth of anaerobic bacteria. (Turan and Kocatepe 2013). In food microbiology, the first bacterial species that come to mind when anaerobic bacteria are mentioned are Clostridium species. Apart from these, other anaerobic or facultative anaerobic bacteria can be found in foods. Coliform group bacteria and *Staphylococcus aureus* can also grow in Reinforced Clostridial Agar medium (Halkman 2005).

TAB counts of all samples are shown in Fig. 1.E. The TAB count of the nanoemulsion was determined to be < 0.30 log CFU/g. In the first 6 days of storage, the number of TABs was < 0.30 log CFU/g in all groups. Increases in TAB numbers during storage were first observed in the raw groups (RT and RTL). On the 21st day of storage, the TAB counts of RT, RTL, ST and STL groups were determined as 6.18 log CFU/g, 3.68 log CFU /g, 3.25 log CFU /g and 3.11 log CFU/g, respectively. At the end of storage, these values increased to 5.04 log CFU/g, 4.99 log CFU/g and 4.47 log CFU/g in RTL, ST and STL groups, respectively. Turan and Kocatepe (2013) reported that total anaerobic bacterial growth of sea bass fish vacuum packed and stored under refrigerator conditions started on the 12th day of storage. In our study, it was observed that anaerobic bacteria growth started on the 9th day in the control group. It can be said that the difference may be due to fish species, ambient and environmental conditions and processing processes. There is no literature on the monitoring of anaerobic bacterial development in nanoemulsified products during storage. The steaming and nanoemulsion processes used in this study, when considered together, show that nanoemulsions can be used to improve food quality and extend shelf life by retarding anaerobic microbial growth (p˂0.05).

### Total volatile base nitrogen (TVB-N)

**Fig. 2:**
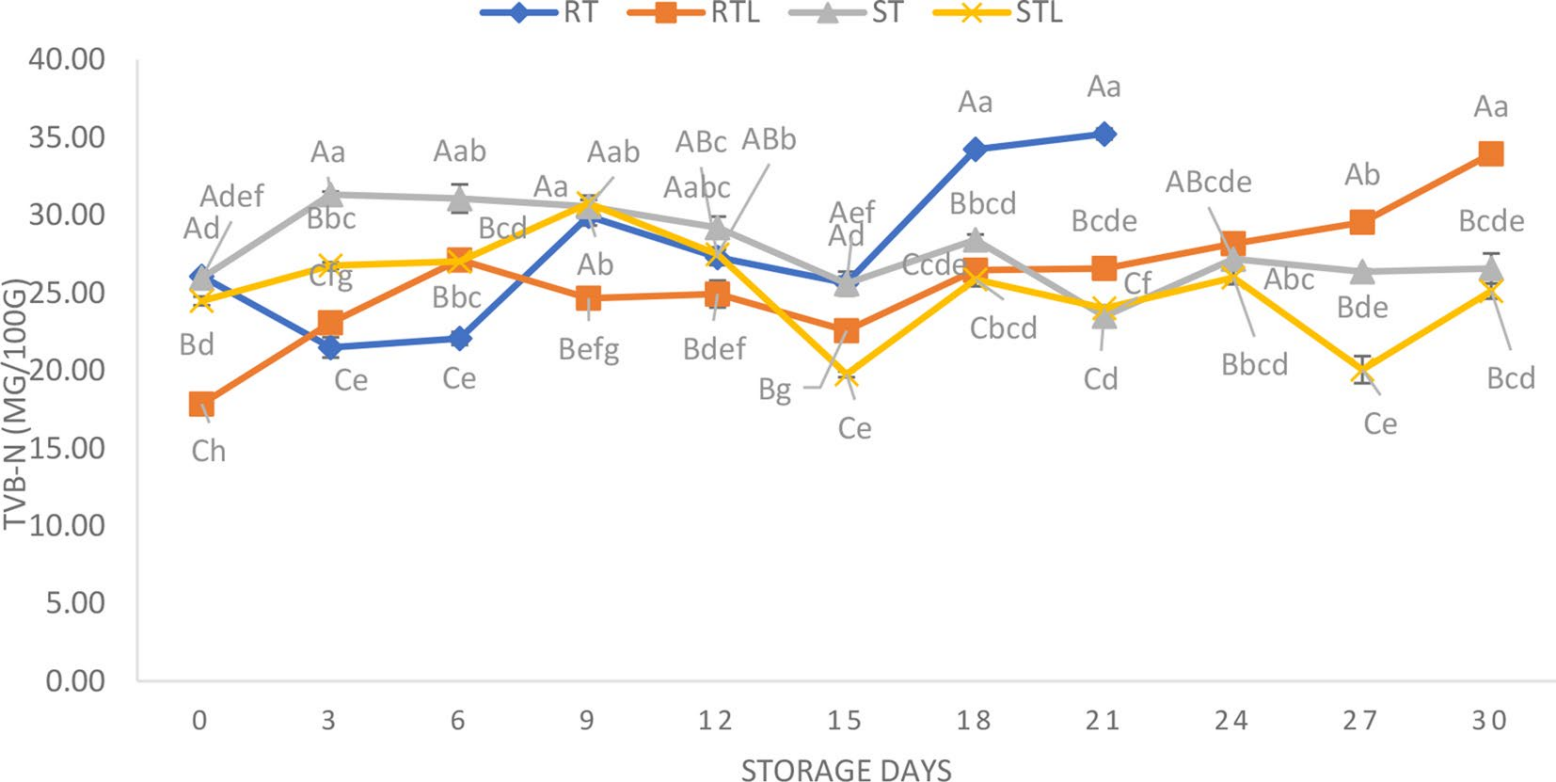
TVB-N values of raw or steamed trout cubes treated/not treated with lemon oil nanoemulsion **RT**: Raw trout cubes, **RTL**: Raw trout cubes treated with lemon oil nanoemulsion, **ST**: Steamed trout cubes, **STL**: Steamed trout cubes treated with lemon oil nanoemulsion

The TVB-N shown in Fig. 2 was evaluated to find out the quality changes of nanoemulsion treated fish meat. In this regard, the initial TVB-N contents of raw trout cubes (RT), the lemon oil nanoemulsion treated raw trout cubes (RTL), steamed trout cubes (ST), and finally the steamed trout cubes treated with lemon oil (STL) were found as 26.06, 17.84, 25.95 and 24.47 mg/100 g, respectively. Initial results showed that applying a nanoemulsion to the raw fish cubes before the cooking significantly delayed the TVB-N acceleration (*p* < 0.05). This is an extremely significant result for the raw materials to be processed. Furthermore, a slight increase in TVB-N acceleration of RTL samples was observed upon 21 days of storage, and this increase was found to be slower than that of RT (untreated crude control) samples. For the cooked samples, an increase was observed with time until the 9th day of cold storage. However, due to of the fact that there may be an interaction between the release profile of the nanoemulsion depending on time and cooking process, a significant decrease (ST: 30.56 to 23.49 and STL: 30.76 to 19.75) was observed within 21 days. On the last day of storage, the TVB-N content of RTL, ST and STL groups were found as 33.94, 26.56 and 25.11 mg/100 g, respectively. All groups remained within consumption limits. It is thought that the reason for the relatively lower TVB-N values in groups C and D compared to group B in the present study may be due to the combined effect of steaming for groups C and D and the combined effect of steaming and lemon oil nanoemulsion for group D.

In this sense, when the TVB-N results were evaluated in the context of the potential effect of nanoemulsion on raw material and cooking process, this application provided higher protection for raw material and steamed samples treated with nanoemulsion. Nanotechnology applications in food science have recently been able to provide high efficiency, such that, for 11 days of storage, it was revealed in sea bream fillets that control samples exceeded 51.65 mg/100 g TVB-N value, and the samples coated with nanofibers possessed 34.61 mg/100 g TVB-N value (Ceylan et al. 2017). Furthermore, as shown in a nanotechnology based study by Khanipour et al. (2020), a relationship was found between the microbiological growth and the TVB-N amount of fish fillets. Furthermore, nano-composite clay/LDPE film limited the rapid TVB-N accumulation in fish fillets stored at refrigerated conditions. In this regard, a similar case was reported in the present study. Nanogelatin coating with thymol and nisin delayed TVB-N increase in raw fish meat samples stored at 4 °C during the shelf life (Hashemi et al. 2022). As several recent studies suggest, some nanotechnology approaches to raw materials provided better quality. With the present study, nanoemulsion treatment promised a novel strategy to limit the rapid TVBN increase, especially in cooked samples.

### Sensory evaluation

Lemon essential oil is known to have attractive organoleptic properties (Nunes et al. 2021). It was reported that the application of lemon flavor to fish and fish products is acceptable, and, therefore, the addition of lemon essential oils to these products would have positive results (Alfonzo et al. 2017). In addition, nanoemulsion-based essential oils are known to have positive effects on sensory quality by eliminating fishy odor (Durmus 2020).

The sensory evaluation of raw and cooked trout cube samples is shown in Table 1. Regarding the overall acceptability values, the RT group was considered as non-consumable on the 9th day of storage, while the RTL group was considered as non-consumable on the 15th day of storage. In this sense, it can be said that the nanoemulsion treated with lemon oil extends the shelf life of raw trout cubes by 3 days. However, looking at the overall acceptability scores when comparing the raw (RT- RTL) and cooked (SRT-SRTL) groups; the longest shelf life was found to be 15 days, with nanoemulsion containing lemon oil applied and steamed trout after raw storage (SRTL) (*p* < 0.05). Considering the overall acceptability scores of only the cooked groups; it was found that the STL group was the most desirable group with a shelf life of 27 days. The STL group was followed by the ST, SRTL and SRT groups with a shelf life of 24, 15 and 9 days, respectively. The sensory evaluation examined whether applying a nanoemulsion before or after cooking the trout would be more effective. Accordingly, it was observed that the group that was first cooked and then treated with nanoemulsion had a longer shelf life but was rated higher in terms of appearance, smell, taste, texture and overall acceptability.

**Table 1:** Sensory analysis results of Raw and steamed fish cubes

		Raw		Raw-Steamed				Steamed			
	Days	RT	RTL	RT	RTL	SRT	SRTL	SRT	SRTL	ST	STL
**Appearance**	0	5.00 ± 0.00^Xx^	5.00 ± 0.00^Xx^	5.00 ± 0.00α^α^	5.00 ± 0.00α^α^	5.00 ± 0.00α^α^	5.00 ± 0.00α^α^	5.00 ± 0.00^Aa^	5.00 ± 0.00^Aa^	5.00 ± 0.00^Aa^	5.00 ± 0.00^Aa^
	3	5.00 ± 0.00^Xx^	4.70 ± 0.10^Yx^	5.00 ± 0.00α^α^	4.70 ± 0.10α^α^	4.80 ± 0.09α^α^	4.80 ± 0.09αα^β^	4.80 ± 0.09^Aa^	4.80 ± 0.09^Aab^	4.88 ± 0.07^Aa^	4.95 ± 0.03^Aa^
	6	3.40 ± 0.18^Xy^	3.80 ± 0.17^Xy^	3.40 ± 0.18β^β^	3.80 ± 0.17β^β^	3.60 ± 0.11β^β^	4.40 ± 0.11α^β^	3.60 ± 0.11^Bb^	4.40 ± 0.11^Ab^	4.35 ± 0.14^Ab^	4.60 ± 0.11^Aabc^
	9	2.60 ± 0.11^Xz^	2.75 ± 0.09^Xz^	2.60 ± 0.11β^γ^	2.75 ± 0.09β^γ^	2.80 ± 0.09β^γ^	3.30 ± 0.10α ^γ^	2.80 ± 0.09^Cc^	3.30 ± 0.10^Bc^	4.38 ± 0.11^Ab^	4.70 ± 0.10^Aab^
	12	2.15 ± 0.16^Yz^	2.65 ± 0.11^Xzw^	2.15 ± 0.16α^γ^	2.65 ± 0.11α^γδ^	2.30 ± 0.13α^δ^	2.55 ± 0.16α^δ^	2.30 ± 0.13^Bd^	2.55 ± 0.16^Bd^	4.01 ± 0.01^Ab^	4.30 ± 0.09^Abcd^
	15	-	2.60 ± 0.11^zw^	-	2.60 ± 0.11α^γδ^	-	2.53 ± 0.11α^δε^	-	2.53 ± 0.11^Bde^	4.00 ± 0.00^Ab^	4.20 ± 0.09^Acd^
	18	-	2.58 ± 0.10^zw^	-	2.58 ± 0.10α^γδ^	-	2.10 ± 0.07β^ε^	-	2.10 ± 0.07^Ce^	3.55 ± 0.11^Bc^	4.15 ± 0.10^Ad^
	21	-	2.20 ± 0.09^w^	-	2.20 ± 0.09^δ^	-	-	-	-	2.80 ± 0.07^Bd^	3.30 ± 0.15^Ae^
	24	-	-	-	-	-	-	-	-	2.73 ± 0.10^Bd^	3.25 ± 0.10^Ae^
	27	-	-	-	-	-	-	-	-	2.30 ± 0.09^Be^	2.95 ± 0.03^Ae^
	30	-	-	-	-	-	-	-	-	1.65 ± 0.11^Af^	1.70 ± 0.10^Af^
**Odor**	0	5.00 ± 0.00^Xx^	5.00 ± 0.00^Xx^	5.00 ± 0.00α^α^	5.00 ± 0.00α^α^	5.00 ± 0.00α^α^	5.00 ± 0.00α^α^	5.00 ± 0.00^Aa^	5.00 ± 0.00^Aa^	5.00 ± 0.00^Aa^	5.00 ± 0.00^Aa^
	3	4.90 ± 0.30^Xx^	4.80 ± 0.40^Xx^	4.90 ± 0.30α^α^	4.80 ± 0.40α^α^	5.00 ± 0.00α^α^	4.90 ± 0.20α^α^	5.00 ± 0.00^Aa^	4.90 ± 0.20^Aa^	4.80 ± 0.40^Aa^	4.90 ± 0.20^Aa^
	6	2.60 ± 0.44^Yy^	3.88 ± 0.69^Xy^	2.60 ± 0.44γ^β^	3.88 ± 0.69α^β^	3.15 ± 0.71β^β^	4.00 ± 0.63α^β^	3.15 ± 0.71^Cb^	4.00 ± 0.63^Bb^	4.00 ± 0.00^Bb^	4.60 ± 0.49^Aab^
	9	1.80 ± 0.98^Yz^	2.85 ± 0.45^Xz^	1.80 ± 0.98γ^γ^	2.85 ± 0.45β^γ^	2.13 ± 0.27γ^γ^	3.40 ± 0.49α^γ^	2.13 ± 0.27^Dc^	3.40 ± 0.49^Cc^	3.88 ± 0.27^Bbc^	4.28 ± 0.43^Abc^
	12	-	2.80 ± 0.48^z^	-	2.80 ± 0.48β^γ^	-	3.05 ± 0.15α^γδ^	-	3.05 ± 0.15^Ccd^	3.45 ± 0.52^Bcd^	4.00 ± 0.55^Ac^
	15	-	2.15 ± 0.78^w^	-	2.15 ± 0.78β^δ^	-	2.90 ± 0.66α^δ^	-	2.90 ± 0.66^Bd^	3.10 ± 0.20^Bde^	4.00 ± 0.63^Ac^
	18	-	-	-	-	-	2.00 ± 0.00^ε^	-	2.00 ± 0.00^Ce^	2.95 ± 0.15^Be^	3.90 ± 0.30^Acd^
	21	-	-	-	-	-	-	-	-	2.40 ± 0.49^Bf^	3.50 ± 0.45^Ade^
	24	-	-	-	-	-	-	-	-	2.14 ± 0.69^Bf^	3.20 ± 0.51^Ae^
	27	-	-	-	-	-	-	-	-	2.10 ± 0.83^Af^	2.20 ± 0.24^Af^
	30	-	-	-	-	-	-	-	-	-	2.20 ± 0.56^f^
**Taste**	0	-	-	-	-	-	-	5.00 ± 0.00^Aa^	5.00 ± 0.00^Aa^	5.00 ± 0.00^Aa^	5.00 ± 0.00^Aa^
	3	-	-	-	-	-	-	4.60 ± 0.49^Ab^	4.70 ± 0.40^Aa^	4.80 ± 0.40^Aab^	4.70 ± 0.40^Aab^
	6	-	-	-	-	-	-	3.45 ± 0.47^Bc^	3.80 ± 0.40^Bb^	4.40 ± 0.72^Ab^	4.60 ± 0.49^Aab^
	9	-	-	-	-	-	-	2.20 ± 0.37^Dd^	3.05 ± 0.15^Cc^	3.63 ± 0.47^Bc^	4.25 ± 0.51^Abc^
	12	-	-	-	-	-	-	-	2.40 ± 0.49^Bd^	3.55 ± 0.57^Ac^	3.88 ± 0.45^Acd^
	15	-	-	-	-	-	-	-	2.35 ± 0.39^Cd^	3.35 ± 0.48^Bc^	3.80 ± 0.40^Acde^
	18	-	-	-	-	-	-	-	2.00 ± 0.00^Ce^	3.30 ± 0.40^Bc^	3.77 ± 0.41^Ade^
	21	-	-	-	-	-	-	-	-	2.10 ± 0.20^Bd^	3.35 ± 0.67^Aef^
	24	-	-	-	-	-	-	-	-	2.05 ± 0.22^Bd^	2.93 ± 0.23^Af^
	27	-	-	-	-	-	-	-	-	1.95 ± 0.22^Bd^	2.30 ± 0.46^Ag^
	30	-	-	-	-	-	-	-	-	-	1.40 ± 0.49^h^
**Texture**	0	5.00 ± 0.00^Xx^	5.00 ± 0.00^Xx^	5.00 ± 0.00α^α^	5.00 ± 0.00α^α^	5.00 ± 0.00α^α^	5.00 ± 0.00α^α^	5.00 ± 0.00^Aa^	5.00 ± 0.00^Aa^	5.00 ± 0.00^Aa^	5.00 ± 0.00^Aa^
	3	4.75 ± 0.09^Yx^	5.00 ± 0.00^Xx^	4.75 ± 0.09αβ^α^	5.00 ± 0.00α^α^	4.60 ± 0.11β^α^	4.80 ± 0.09αβ^α^	4.60 ± 0.11^Aa^	4.80 ± 0.09^Aa^	4.80 ± 0.09^Aa^	4.80 ± 0.09^Aab^
	6	2.95 ± 0.03^Yy^	3.35 ± 0.12^Xy^	2.95 ± 0.03γ^β^	3.35 ± 0.12βγ^β^	3.45 ± 0.11β^β^	4.20 ± 0.17α^β^	3.45 ± 0.11^Bb^	4.20 ± 0.17^Ab^	4.65 ± 0.16^Aa^	4.55 ± 0.13^Aabc^
	9	2.60 ± 0.30^Xy^	3.23 ± 0.17^Xy^	2.60 ± 0.30αβ^β^	3.23 ± 0.17α^β^	2.43 ± 0.16β^γ^	3.20 ± 0.09α^γ^	2.43 ± 0.16^Dc^	3.20 ± 0.09^Cc^	4.03 ± 0.02^Bb^	4.55 ± 0.09^Aabc^
	12	-	2.70 ± 0.09^z^	-	2.70 ± 0.09β^γ^	-	3.15 ± 0.07α^γ^	-	3.15 ± 0.07^Bc^	4.00 ± 0.14^Ab^	4.30 ± 0.22^Abc^
	15	-	2.55 ± 0.13^z^	-	2.55 ± 0.13β^γ^	-	3.00 ± 0.17α^γ^	-	3.00 ± 0.17^Cc^	3.60 ± 0.11^Bbc^	4.30 ± 0.09^Abc^
	18	-	2.50 ± 0.11^z^	-	2.50 ± 0.11α^γ^	-	2.40 ± 0.08α^δ^	-	2.40 ± 0.08^Cd^	3.50 ± 0.10^Bc^	4.15 ± 0.10^Acd^
	21	-	2.25 ± 0.11^z^	-	2.25 ± 0.11α^γ^	-	2.00 ± 0.00β^δ^	-	2.00 ± 0.00^Cd^	2.75 ± 0.09^Bd^	3.65 ± 0.12^Ad^
	24	-	-	-	-	-	-	-	-	2.15 ± 0.07^Be^	2.90 ± 0.07^Ae^
	27	-	-	-	-	-	-	-	-	1.80 ± 0.09^Be^	2.80 ± 0.09^Ae^
	30	-	-	-	-	-	-	-	-	-	1.45 ± 0.11^f^
**Overall acceptability**	0	5.00 ± 0.00^Xx^	5.00 ± 0.00^Xx^	5.00 ± 0.00α^α^	5.00 ± 0.00α^α^	5.00 ± 0.00α^α^	5.00 ± 0.00α^α^	5.00 ± 0.00^Aa^	5.00 ± 0.00^Aa^	5.00 ± 0.00^Aa^	5.00 ± 0.00^Aa^
	3	5.00 ± 0.00^Xx^	5.00 ± 0.00^Xx^	5.00 ± 0.00α^α^	5.00 ± 0.00α^α^	4.70 ± 0.10β^α^	4.70 ± 0.10β^α^	4.70 ± 0.10^Aa^	4.70 ± 0.10^Aa^	4.90 ± 0.00^Aab^	4.90 ± 0.00^Aa^
	6	2.85 ± 0.11^Yy^	3.70 ± 0.17^Xy^	2.85 ± 0.11α^β^	3.70 ± 0.17αβ^β^	3.35 ± 0.07β^β^	4.10 ± 0.15α^β^	3.35 ± 0.07^Cb^	4.10 ± 0.15^Bb^	4.55 ± 0.13^ABb^	4.58 ± 0.12^Aab^
	9	1.95 ± 0.19^Yz^	2.85 ± 0.08^Xz^	1.95 ± 0.19β^γ^	2.85 ± 0.08α^γ^	2.80 ± 0.15α^γ^	3.30 ± 0.07α^γ^	2.80 ± 0.15^Dc^	3.30 ± 0.07^Cc^	3.90 ± 0.04^Bc^	4.30 ± 0.11^Abc^
	12	-	2.76 ± 0.04^z^	-	2.76 ± 0.04β^γ^	-	3.11 ± 0.04α^γ δ^	-	3.11 ± 0.04^Bcd^	3.83 ± 0.11^Ac^	4.20 ± 0.15^Abc^
	15	-	1.86 ± 0.10^w^	-	1.86 ± 0.10β^δ^	-	2.92 ± 0.05α^δ^	-	2.92 ± 0.05^Cd^	3.75 ± 0.07^Bc^	4.10 ± 0.09^Acd^
	18	-	-	-	-	-	-	-	-	3.20 ± 0.09^Bd^	4.05 ± 0.03^Acd^
	21	-	-	-	-	-	-	-	-	2.75 ± 0.09^Be^	3.70 ± 0.11^Ad^
	24	-	-	-	-	-	-	-	-	2.35 ± 0.10^Bf^	3.00 ± 0.09^Ae^
	27	-	-	-	-	-	-	-	-	1.90 ± 0.07^Bg^	2.50 ± 0.11^Af^
	30	-	-	-	-	-	-	-	-	-	1.50 ± 0.10^g^

**RT**: Raw trout cubes, **RTL**: Raw trout cubes treated with lemon oil nanoemulsion, **SRT**: Steamed trout cubes after raw storage, **SRTL**: Trout cubes treated with lemon oil nanoemulsion and steamed after raw storage, **ST**: Steamed trout cubes, **STL**: Steamed trout cubes treated with lemon oil nanoemulsion

**X**,** Y**,** Z. α**,** β**,** γ…A**,** B**,** C**,**.→** Capital letters indicate the difference between groups on the same day (*p* < 0.05)

**x**,** y**,**z…α**,** β**,** γ… a**,** b**,**c…↓** Lowercase letters represent the difference between days within the same group (*p* < 0.05)

**α**,** β**,** γ…**Exponential expressions are lowercase and others are uppercase

Durmus (2020) determined the sensory shelf life of the nanoemulsion treated with lemon oil on raw trout to be 14 days. This result was close to the sensory shelf life (12 days) of the raw trout treated with the lemon oil nanoemulsion that we used in our study. In another study, microemulsion lemon essential oil was added to salted sardines and the highest sensory scores were recorded for flavor and overall acceptability in the presence of lemon essential oil (Alfonzo et al. 2017). On the other hand, in our present study, the sensory shelf life was 24 days for steam-cooked samples according to the overall acceptance criterion, while this period increased to 27 days for both steam-cooked and lemon-oil-treated nanoemulsion-applied samples. It was observed that the combined effect of steaming and nanoemulsion application extended the sensory shelf life of the product by 3 days.

It was determined that the group cooked first and then nanoemulsified (STL) had a longer shelf life and was evaluated with higher scores in terms of appearance, odor, taste, texture and general acceptability criteria. This is thought to be the result of cooking fresh fish meat without waiting and exposing it directly to the preservative effect of lemon oil nanoemulsion. Therefore, fish meat was processed without being affected by spoilage parameters and without increased microbial and enzymatic activities. Therefore, taking this issue into consideration in combined applications using heat treatment and nanoemulsion in seafood processing technologies will contribute to future studies. However, realization of such applications in the seafood processing sector may require new infrastructure and equipment. The adaptation of food or seafood processing technologies to nanotechnology requires more time and extensive research.

### Principle component analysis (PCA)

**Fig. 3:**
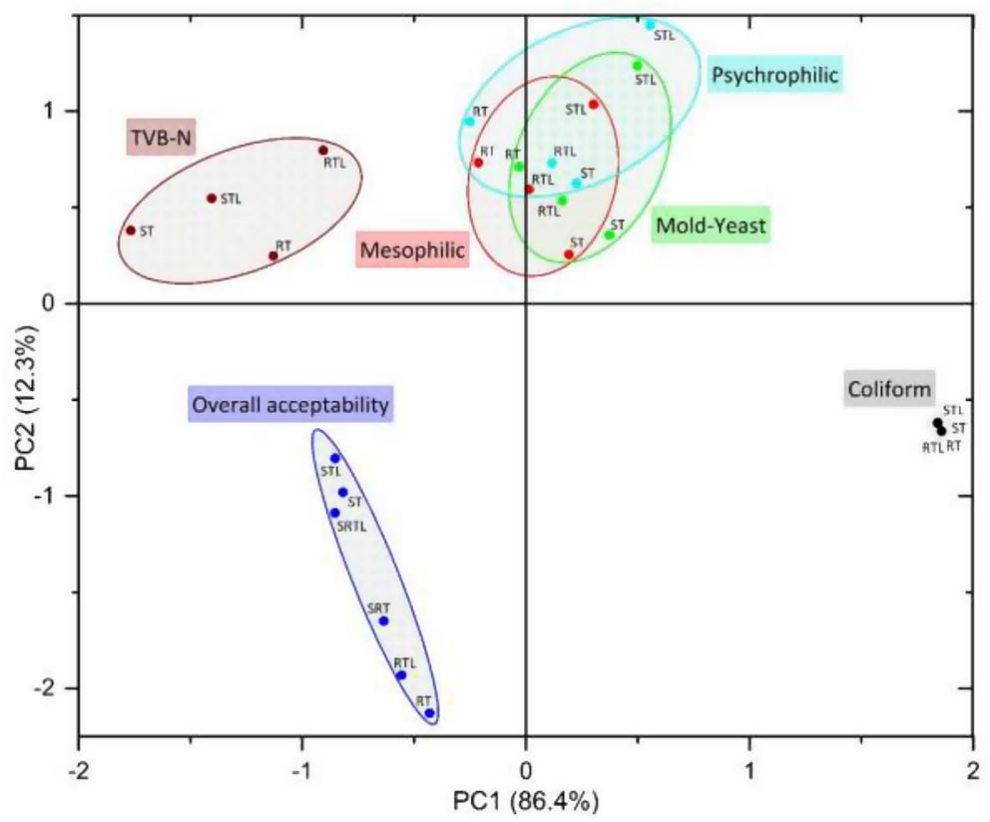
Principle component analysis (PCA) score plots results. **RT**: Raw trout cubes, **RTL**: Raw trout cubes treated with lemon oil nanoemulsion, **SRT**: Steamed trout cubes after raw storage, **SRTL**: Trout cubes treated with lemon oil nanoemulsion and steamed after raw storage, **ST**: Steamed trout cubes, **STL**: Steamed trout cubes treated with lemon oil nanoemulsion

PCA was performed with the results of chemical, microbiological and sensory parameters (Fig. 3). The mean centering pre-processing technique was applied to a data set of 26 measurements (Zaragozá et al. 2013). Two principal components accounted for 98.7% of the total variance, of which 86.4% were attributed to PC1 and 12.3% to PC2, indicating that they adequately represented the microbiological (coliform, psychrophilic, mesophilic and yeast-mold), sensorial (overall acceptability) and chemical (TVB-N) changes of the groups during the storage period. TVB-N, overall acceptability, and coliform loads were formed as independent clusters in different areas However, mesophilic and psychrophilic bacterial loads and yeast-mold loads were closely clustered together. In general, most of the same-named groups were closely organized together within the intersecting or overlapping clusters of the analyses, with only a few minor exceptions. Whereas groups with the same name in distant parts of the clusters were also found to be oriented in the same vectorial direction. It can be said that yeast-mold, psychrophilic, and mesophilic changes displayed very similar behaviors. In the PCA analysis conducted by Calanche et al. (2013) with salmon (*Salmo salar*), similar patterns and distributions were observed for mesophilic bacterial load, TVB-N values, and sensory analysis scores. When the results of the microbiological analysis (Fig. 1) and the PCA graph are interpreted together, it can be confirmed that ST and STL are the groups with the least microbiological load change over time in overlapping clusters. In the coliform load, it is expected to overlap since the load is similar in all groups except for the last day. When the overall acceptability values are evaluated in the sensory analysis, it is possible to say that the groups at the top of the cluster (STL, ST, SRTL) are slightly more differentiated than the other three groups at the bottom (Table 1).

## Conclusion

In this study, a homogeneous and well-stabilized nanoemulsion was obtained using fabricated lemon oil. It can be said that raw samples treated with nanoemulsion are generally in better condition than the control group in terms of the TMAB TPAB, TYM and TCB counts during storage. In addition, when all groups were evaluated together, the samples treated with steaming and nanoemulsion were in better condition in terms of both microbial and TVB-N values, clearly proving the combined effect of both applications on fish meat. The overall sensory acceptability values showed that the control sample (RT) and the RTL had a shelf life of 6 and 12 days, respectively. However, steaming extend the shelf life by 3 days for the same samples. The longest shelf life was recorded in the samples treated with lemon oil nanoemulsion after steaming (STL), as found to be 27 days. The present results were also confirmed by PCA analysis.

As a result, it was observed that the combined effect of steam treatment and nanoemulsion application in ready-to-eat trout samples had a very positive effect on the safety and shelf life of the product. Existing processes that can be applied in seafood processing and the food industry also have the potential to generate new research and contribute to the industry by improving them through various cooking techniques and various nanoprocessing applications. In addition, in future studies on nanoemulsion and fish preservation, it may be recommended to try nanoemulsion applications containing different fish species/ crustaceans and different oils with different cooking techniques.

## Data Availability

The corresponding author will provide the created datasets during the current investigation, upon reasonable request.
